# Characterization of green-synthesized zinc oxide nanoparticles and its influence on post-harvest shelf-life of garlic against black mold disease caused by *Aspergillus niger*

**DOI:** 10.3389/fmicb.2025.1532593

**Published:** 2025-02-13

**Authors:** Govind Kumawat, Deepak Rajpurohit, Divya Vyas, Ali Asger Bhojiya, Sudhir Kumar Upadhyay, Devendra Jain

**Affiliations:** ^1^Department of Molecular Biology and Biotechnology, Rajasthan College of Agriculture, Maharana Pratap University of Agriculture and Technology, Udaipur, India; ^2^Department of Processing and Food Engineering, College of Technology and Engineering, Maharana Pratap University of Agriculture and Technology, Udaipur, India; ^3^Department of Botany, U.S. Ostwal P.G. College, Mohanlal Sukhadia University, Chittorgarh, Rajasthan, India; ^4^Academy of Biology and Biotechnology, Southern Federal University, Rostov-on-Don, Russia; ^5^Research and Development Cell, Lovely Professional University, Phagwara, Punjab, India

**Keywords:** *Aspergillus niger*, confirmatory test, green synthesis, molecular identification, post-harvest disease management, ZnO-NPs

## Abstract

Garlic is an important spice crop used for flavoring food and has a long history of use in traditional medicine. However, black mold is a common fungal disease affecting garlic, which was caused by an *Aspergillus* infection. This disease significantly impacts both the production and quality of garlic. Therefore, this study aimed to evaluate the antifungal activity of novel green-synthesized zinc oxide nanoparticles (ZnO-NPs) against black mold diseases in garlic. An environmentally friendly green synthesis technique was used to produce ZnO-NPs using zinc-tolerant bacteria *Serratia* sp. (ZTB24). In the present study the experimental analysis viz. UV-Vis spectroscopy at 380 nm, transmission electron microscopy (TEM), dynamic light scattering (DLS), and zeta potential confirmed the successful biosynthesis of green ZnO-NPs from *Serratia* sp. The poisoned food technique and spore germination test revealed the antifungal activities of ZnO-NPs against *A. niger* under *in vitro* conditions. The presence of disease-causing *A. niger* fungus was confirmed through its isolation from infected garlic bulbs, and it was further identified at the molecular level using inter-transcribed sequence (ITS) rDNA sequencing. ZnO-NPs reduced the mycelial growth up to 90% and the 73% spore germination at 250 μg ml^−1^ concentration of ZnO-NPs. The ZnO-NPs were further used *in vivo* at different concentrations (50, 100, 250, and 500 ppm) in the post-harvest treatment of garlic. The percentage of disease severity was assessed after 7 and 14 days, and the application of 500 ppm of ZnO-NPs exhibited 0% disease severity in the pre-inoculation method, while disease severity of black mold disease in garlic plant was recorded at 1.10% after 7 days and 0.90% after 14 days in the post-inoculation method, compared to the control group. Hence, the antifungal activity of ZnO-NPs synthesized using the green technique paves the way for the development of natural fungicides, offering a sustainable and renewable alternative to traditional chemical control methods.

## 1 Introduction

Garlic (*Allium sativum* L.), a significant spice crop belonging to the Alliaceae family, is the second most frequently grown crop in the family after onions (Nainwal et al., 2014). Compared to other bulb crops, it has a higher nutritional value and is abundant in carbohydrates, proteins, minerals such as phosphorus, potassium, calcium, and magnesium. Green garlic has a very high ascorbic acid concentration (Srivastava et al., 2012; Pacholczyk-Sienicka et al., 2024). Garlic has been used historically to improve the flavor of food in various cultures. Additionally, it is used in traditional medicine and valued for its aromatic properties (Arreola et al., 2015). In terms of both acreage and global garlic production, India ranks second to China, with a production of 3.7 million tonnes in 2023–2024, and the Spices Board reveals that exports reached 56,823 tonnes for the same period (Sonwani et al., 2024).

Garlic can be severely affected by fungal diseases, such as Phylium blight, *Colletotrichum* blight, *Fusarium* basal rot, white rot (sclerotia rot), black mold (*Aspergillus* spp.), blue mold (*Penicillium* spp.), purple blotch, and damping-off. Black mold harms garlic bulbs both in the field and in storage conditions (Singh et al., 2021). This disease is typically caused by an infection from *Aspergillus niger* in stored post-harvest garlic bulbs and accounts for 30–40% losses during storage (Xie et al., 2023; Gaur and Chandel, 2023). Garlic growers and exporters can gain a competitive advantage in global markets by developing alternative post-harvest disease management strategies that replace the current synthetic fungicides with more consumer-friendly and environmentally sustainable alternatives. One such substitute measure is the application of nanomaterials that can significantly reduce the post-harvest economic losses in garlic. The broad range of applications of nanomaterials in agriculture, electronics, imaging, catalysis, chemistry, energy, and medicine led to an increase in its commercial demand. Nanomaterials differ from traditional materials due to their unique properties, which include large surface areas, ultra-small particle sizes (between 1 and 100 nm), and different physio-chemical and morphological characteristics (Purohit et al., 2022). The advancement in nanotechnology has enabled the synthesis of nanoparticles with potential applications in electronics, textiles, medicine, agriculture, and treatments (Jain et al., 2009). Antibacterial, antifungal, and anticancer properties of ZnO-NPs make it a vital tool for many research-based experiments (Gao et al., 2019).

It is commonly known that certain metallic nanoparticles, particularly ZnO-NPs, can suppress filamentous fungi and can be used as a new class of fungicides. The food sector is drawn to ZnO-NPs due to their distinct physicochemical and biological characteristics, which position them as a promising antifungal agent. The antifungal qualities and manufacturing techniques of ZnO-NPs were examined in several recent studies, and potential antifungal pathways were investigated (Sun et al., 2018; Sharma et al., 2023). Due to their unique properties, which include stable chemical nature, non-toxic characteristics, UV and antibacterial protection, strong photocatalytic activity, and excellent visible wavelength spectrum clarity, ZnO-NPs are among the metal oxides used to treat materials (Karthik et al., 2017). The objective of the present study was threefold: (i) the isolation and identification of pathogenic fungi, (ii) the green synthesis of ZnO-NPs, and (iii) the assessment of the antifungal activity of green-synthesized ZnO-NPs against *A. niger*-mediated garlic (*in vitro* and *in vivo* conditions).

## 2 Materials and methods

### 2.1 Collection of disease sample

During the Rabi season, garlic samples were collected from the Hadoti region (which includes Kota, Bundi, Baran, and Jhalawar districts of Rajasthan, India) that showed signs of disease, such as black powdery masses on the outer surface and water-soaked areas developing between the outer flaky skin and the bulb’s initial fleshy scales. These symptomatic garlic cloves were brought to the laboratory for pathogen isolation and subsequent analysis.

### 2.2 Fungal isolation, pathogenicity test, and identification

The fungal pathogen was isolated from the infected garlic clove using the method outlined by Ahmed et al. (2022). A diseased portion was surface-sterilized with mercuric chloride (1:1000 for 20 s), followed by three washes with sterile water. The specimen was then cut into small pieces, including a portion of healthy tissue, and placed on potato dextrose agar (PDA) petri-plates under aseptic conditions. The plates were incubated at a temperature of 27 ± 2°C. The fungal growth appeared in 4–5 days after incubation and mycelial color turned yellow to brownish-black at the center. Sub-cultures from uncontaminated periphery growth using the hyphal tip culture method were made on PDA plates and slants to obtain purified culture. A seven-day-old purified culture was then used for pathogenicity testing by applying Koch’s postulates. Pathogenicity tests were performed in the laboratory on fresh garlic bulbs by spraying the pathogenic fungal spore suspension on fresh garlic cloves. Disease symptoms were assessed after 7 days from the inoculation. Isolated fungus having pathogenicity was further identified from the Indian Type Culture Collection, New Delhi.

### 2.3 Molecular characterisation of fungus

The isolated fungal pathogen culture was maintained on PDA at 27 ± 2°C and the mycelia grown in liquid PDA media were used for DNA extraction. The mycelia were used for isolating genomic DNA (Lücking et al., 2020). The genomic DNA isolated from fungal pathogen was run on agarose gel to check its purity and quantity by standard procedures. Polymerase chain reaction (PCR) amplification by PCR Primers (Inter Transcribed Sequence 1 and Inter Transcribed Sequence 4) was performed following the methodologies in literature (White et al., 1990). The amplified product was further gel-purified and sequenced at Triyat Biosciences, Nagpur, India. The sequenced data were compared with the nucleotide-Basic Local Alignment Search Tool (BLASTn) at National Center for Biotechnology Information (NCBI) for phylogenetic analysis (Jain et al., 2020).

### 2.4 Green synthesis of ZnO-NPs and its characterisation

An eco-friendly green synthesis technique was employed for developing ZnO-NPs by using novel zinc-tolerant bacteria (ZTB24) for the first time (Jain et al., 2021). The dried ZnO-NP powder was used for characterization and to investigate its potency toward fungal growth inhibition. ZnO-NPs were further characterized by using UV–VIS spectrophotometry (Davaeifar et al., 2019), X-ray diffraction (XRD), dynamic light scattering (DLS), zeta potential, and transmission electron microscopy (TEM) with selected area electron diffraction (SEAD) (Sukhwal et al., 2017).

### 2.5 *In vitro* and *in vivo* antifungal activities of ZnO-NPs against black mold

The antifungal activity of ZnO-NPs was tested against an isolated fungal pathogen by poison food technique at five different concentrations (50 μg/mL, 100 μg/mL, 150 μg/mL, 200 μg/mL, and 250 μg/mL) employing a completely randomized design (CRD), and the radial mycelial growth was compared until full radical growth was observed in the control (without nanoparticles) and the percentage of inhibition was determined using the following formula (Bekker et al., 2006):


$$\begin{array}{l} \mathrm{Inhibition rate}\;\left(\%\right)\\ =\frac{\mathrm{Mycelial growth in control}-\mathrm{Mycelial growth in treatment}}{\mathrm{Mycelial growth in control}}\times 100. \end{array}$$

Furthermore, the inhibition of spore germination against garlic pathogen of ZnO-NPs (50, 100, 150, 200, and 250 μg/mL) was tested following the method described by Sukhwal et al. (2017). The *in vivo* study evaluated the antifungal activities of ZnO-NPs (T1) along with three different commercial formulations, such as SAAF 0.25% (T2), BLITOX 0.25% (T3), Tebuconazole 0.1% (T4), and control (T5) against the isolated fungal pathogen using a CRD design at four different concentrations (50, 100, 250, and 500 ppm), following both pre- and post-inoculation methods. In pre-inoculation, fresh and uniformly sized garlic bulbs were first treated with the selected fungicides and ZnO-NPs at different treatment levels and then inoculated with spore suspension of *A. niger* using the pin pricking method. In post-inoculation, healthy garlic bulbs of uniform size were surface sterilized and then inoculated with spore suspension of *A. niger* by pin pricking method. They were then treated with different concentrations of selected fungicides and ZnO-NPs. The disease severity was recorded on 7th and 14th day after inoculation to determine the best treatment for the management of isolated fungal pathogen:


$$\mathrm{Disease severity}\left(\%\right)=\frac{\mathrm{Area of infected bulb}}{\mathrm{Total area of bulb tissue}}\times 100$$

### 2.6 Statistical analysis

All data were acquired in triplicate and the values expressed as mean ± standard deviation. Data analysis was performed using the analysis of variance (ANOVA) test (Panse and Sukhatme, 1985).

## 3 Results

### 3.1 Fungal isolation and molecular identification

**Figure 1:**
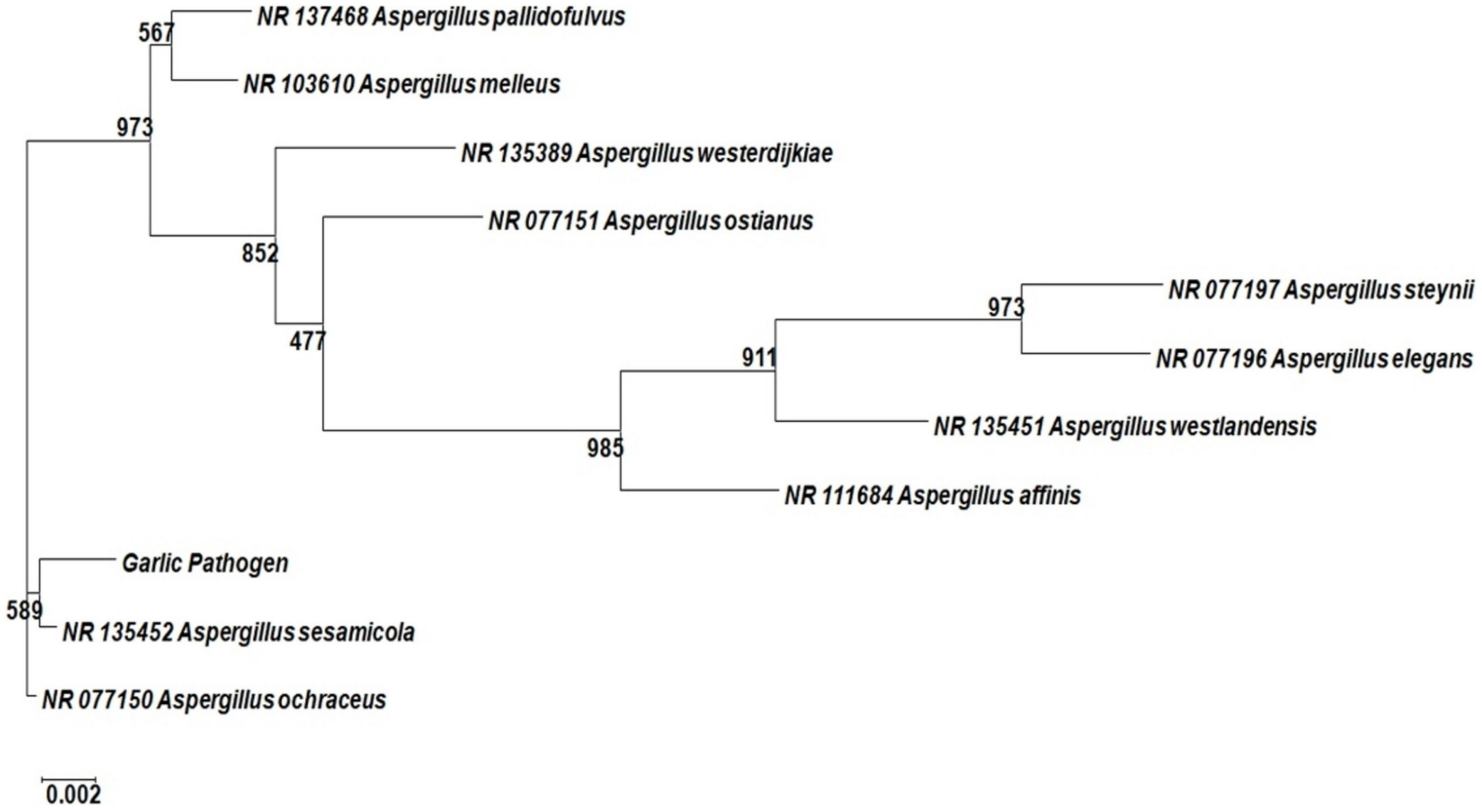
Phylogenetic tree of isolated fungal pathogen causes black mold disease in garlic.

In this investigation, the pathogenic fungus responsible for black mold disease in garlic was identified as *Aspergillus niger* (Identification number IC: 11.717.22). The fungal pathogen’s genomic DNA was extracted, and PCR amplification was performed using ITS1 and ITS4 primers for the amplification of ITS region. The obtained sequences were then compared to older nucleotide BLAST (blastn) sequences, and the closest match was *Aspergillus ochraceus* strain LW3 NCBI accession KT803068 (99.83% similarity), followed by *A. ochraceus* isolate SH0701 NCBI accession JX244861.1, as shown in Figure 1. As a consequence of the similarity in neighbor-joining and maximum-likelihood studies, the isolated strain was identified as an *Aspergillus* spp. Furthermore, the sequencing of the isolated garlic pathogen was submitted to NCBI and the GenBank accession number ON982549 was assigned.

### 3.2 Green synthesis and characterisation ZnO-NPs

Zinc oxide nanoparticles (ZnO-NPs) were produced utilizing a zinc-tolerant bacterial strain, *Serratia marcescens* (ZTB24). The production of ZnO-NPs was qualitatively validated by adding a 0.1 M zinc sulfate solution dropwise to an overnight bacterial culture in equal quantities (14 h at 28°C). The mixture was incubated until the reaction’s color changed from colorless to white, suggesting zinc ion reduction to ZnO nanoparticles. The resultant ZnO-NPs were separated by centrifugation at 14,000 rpm for 10 min and the resultant pellet was washed twice with sterile Milli-Q water and 70% alcohol before drying in an oven at 120°C. The dried ZnO-NPs were then used for further characterization studies.

**Figure 2:**
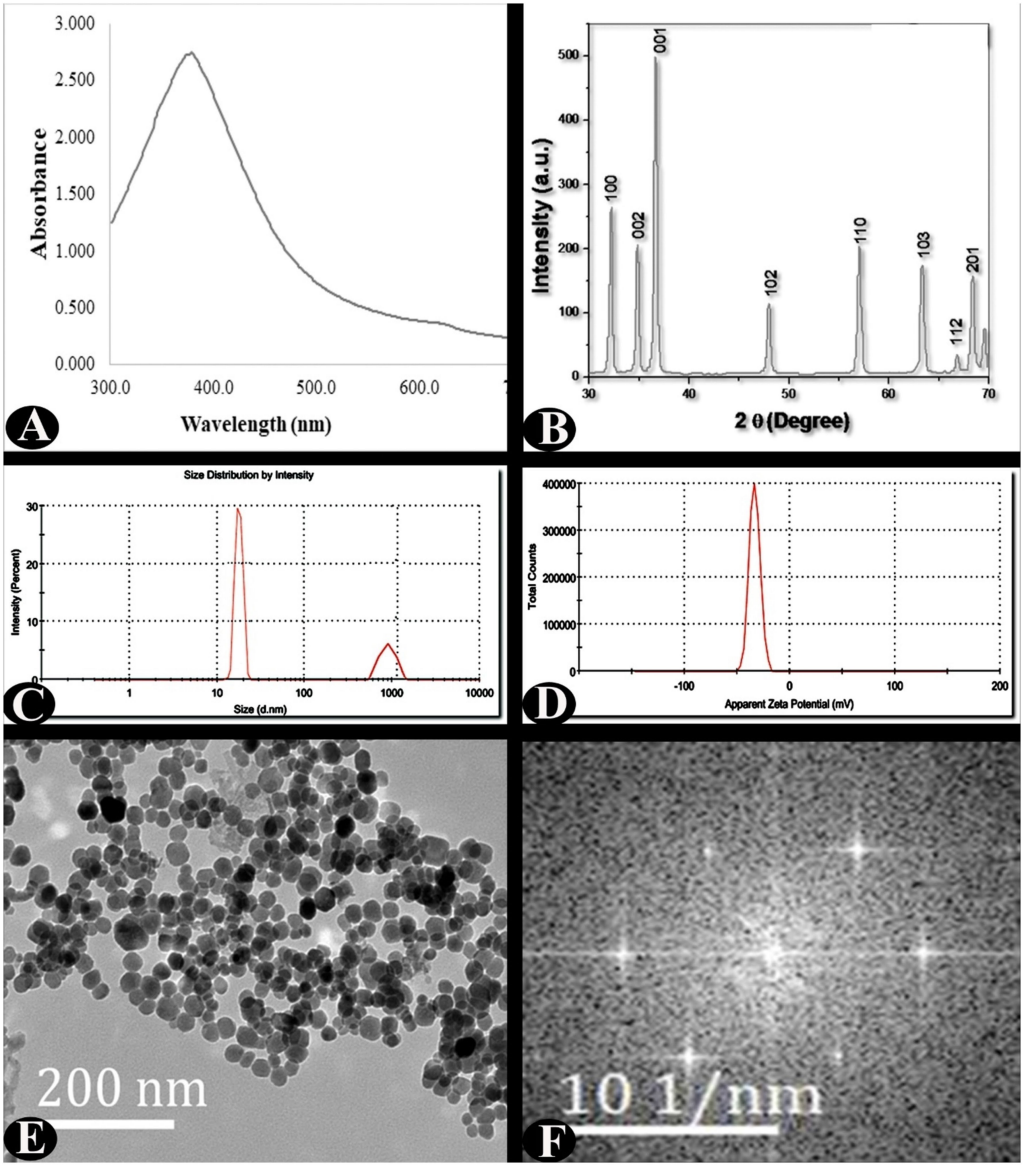
Characterisation of ZnO-NPs synthesised using zinc-tolerant bacteria. **(A)** UV–Vis absorption spectrum. **(B)** X-ray diffraction. **(C)** Particle size determination using dynamic light scattering. **(D)** Zeta potential analysis. **(E)** TEM analysis. **(F)** SAED analysis.

The biologically developed ZnO-NPs were characterized using UV–VIS spectroscopy, XRD, TEM, and SEAD analysis, and DLS with zeta potential (Figure 2). The ZnO nanoparticles have an intense surface plasmon resonance (SPR) ranging between 300 and 600 nm. The UV–visible spectrum of ZnO-NPs exhibited a distinct peak at ~380 nm wavelength, which confirms the formation of ZnO-NPs. The XRD revealed the nanoparticles were pure and crystalline in nature. The peaks at 2θ = 31.7721, 34.4201, 36.2561, 47.5411, 56.6021, 62.8581, 66.3841, 67.9531, 69.0941, 72.5631, and 76.9671 were assigned to the (100), (002), (001), (102), (110), (103), (112), (201) reflection lines of hexagonal ZnO particles, respectively, and our results were comparable to known standard data by the JCPDS (fileno. 043–0002). Furthermore, from the XRD data, the Scherrer method revealed average crystallite size of 27.49 nm. The TEM analysis was used to characterize the shape, morphology, and dimensions of nanoparticles. TEM analysis clearly revealed that ZnO-NPs are poly-dispersed and roughly spherical in shape and their size range was 15–30 nm with mean of 23.09 ± 4.23 nm. The obtained ZnO-NPs were highly crystalline as shown by SAED pattern and SAED spots reflections corresponded to hexagonal crystalline structure. The dynamic light scattering (DLS) and zeta potential were used to characterize the stability, charge of surface, and size distribution of the nanoparticles. The zeta potential value was −33.4 mV, which predicted that the particles are highly stable at room temperature.

### 3.3 Antifungal activities of ZnO-NPs

#### 3.3.1 *In vitro* and *in vivo* studies to evaluate the antifungal activities of ZnO-NPs

##### 3.3.1.1 *In vitro* study

**Figure 3:**
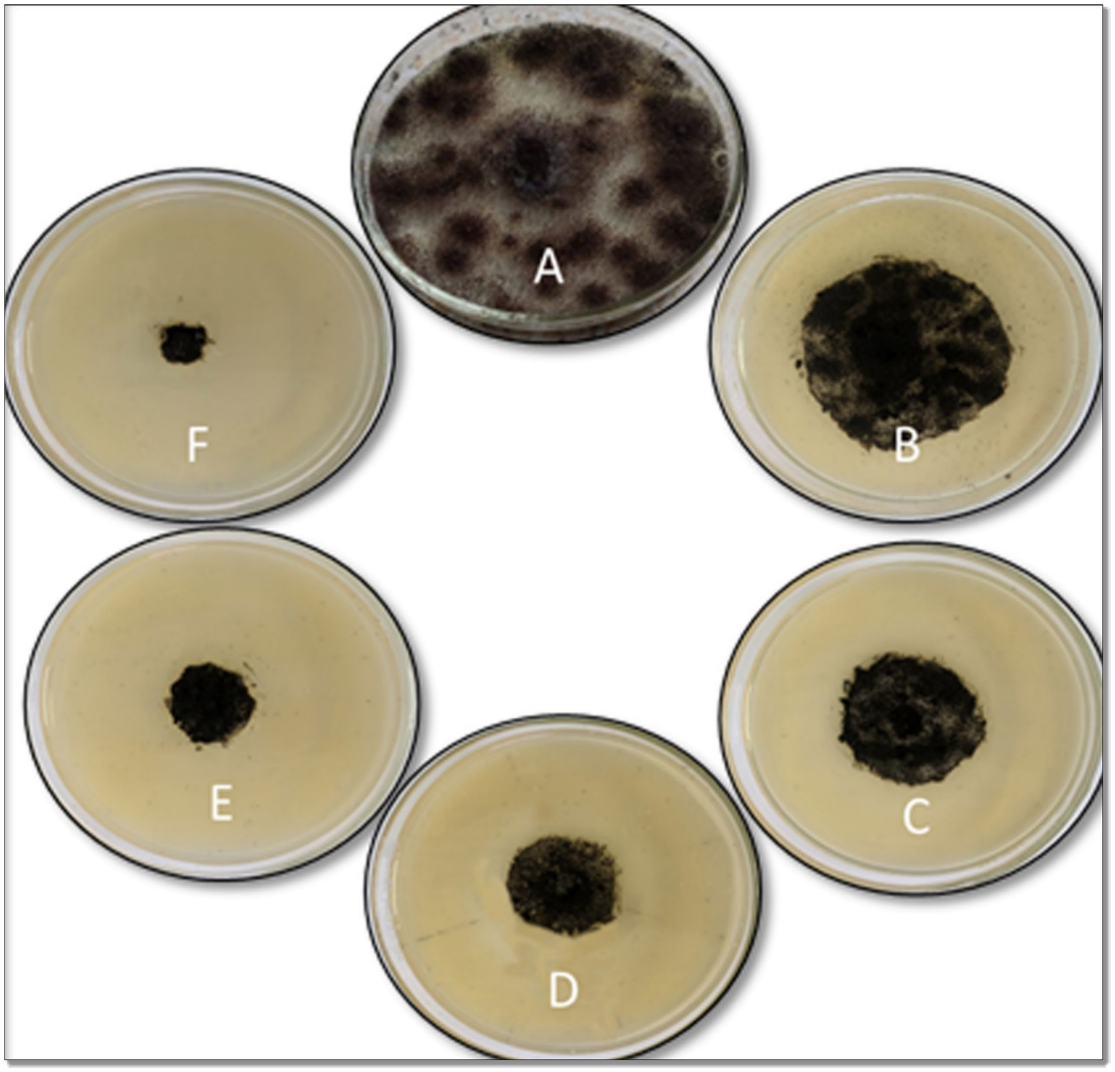
Antifungal activity of ZnO-NPs against *Aspergillus niger* mycelial growth inhibition by poisoned food technique. **(A)** Control. **(B)** 50 μg/mL. **(C)** 100 μg/mL. **(D)** 150 μg/mL. **(E)** 200 μg/mL. **(F)** 250 μg/mL.

The developed ZnO-NPs were investigated for antifungal activities using poisoned food technique and spore germination test. The radial mycelia growth of *A. niger* causing black mold disease in garlic was observed in media containing ZnO-NPs (50, 100, 150, 200, and 250 μg/mL) in a dose-dependent manner (Figure 3). Control without ZnO-NPs was also taken. The experiment plates were incubated at 25°C until full mycelia growth was observed in control. The results are summarized in Table 1. The highest inhibition of mycelia growth of about 90% was observed at the 250 μg/mL concentration of ZnO-NPs. The rate of mycelium growth reduction was directly proportional to the concentration of ZnO-NPs in the medium. Similarly, *A. niger* spore germination was also inhibited in a dose-dependent manner and the results were summarized in Table 1. The highest spore germination inhibition of 73% was observed at 250 μg/mL of ZnO-NPs.

**Table 1:** Effect of different concentrations of ZnO-NPs on *Aspergillus niger.*

Treatments (ZnO-NPs)	Mycelia growth inhibition (%)	Spore germination inhibition (%)
Control	0 ± 0	4.67 ± 1.15
50 μg/mL	37 ± 1.0	5 ± 1.52
100 μg/mL	57 ± 2.40	16 ± 1.52
150 μg/mL	71 ± 0.22	23 ± 2.0
200 μg/mL	83 ± 0.94	40 ± 2.51
250 μg/mL	90 ± 0.40	73 ± 10.01

Data (mean of triplicate value) followed by standard deviation.

##### 3.3.1.2 *In vivo* study

*In vivo* study of ZnO-NPs and three other fungicides, viz., Tebuconazole (0.1%), Blitox (0.25%), and SAAF (0.25%), were tested at four concentrations viz., 50, 100, 250, and 500 ppm (as recommended for commercial formulations) against black mold (*A. niger*) fungus isolated from garlic by pre and post-inoculation methods.

##### 3.3.1.3 Pre-inoculation studies

**Figure 4:**
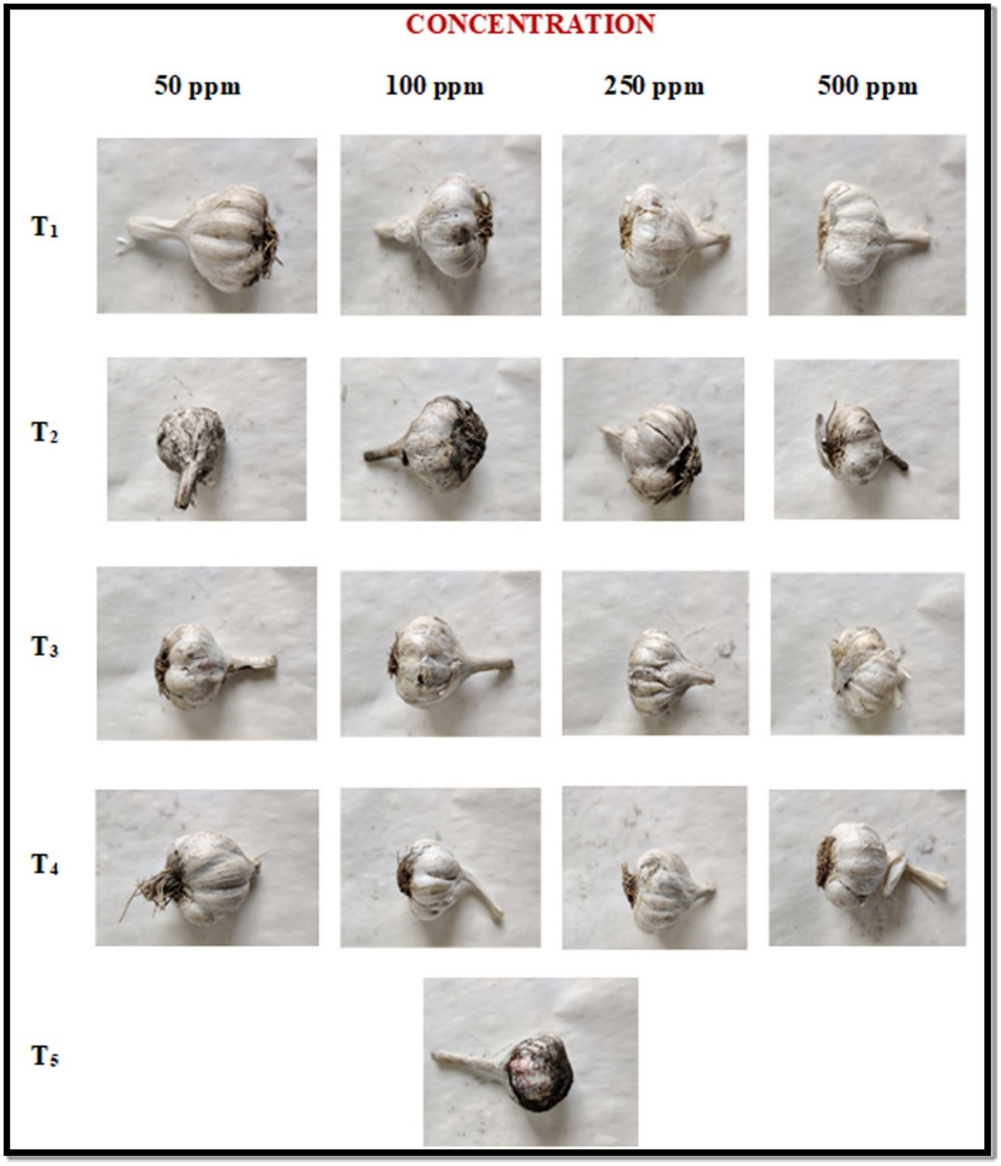
*In vivo* antifungal activities of ZnO-NPs along with commercial formulations to determine black mold bulb disease severity (%) at pre-inoculation conditions. T1: ZnO-NPs; T2: SAAF 0.25%; T3: BLITOX 0.25%; T4: Tebuconazole 0.1%; T5: Control.

In the pre-inoculation method, the garlic bulbs were first treated with ZnO-NP along with the three other fungicides at different concentrations followed by the black mold inoculation and one control was also taken. The percent of disease severity was recorded at 7 and 14 DAI (days after inoculation) and the results are summarized in Table 2 and Figure 4. ZnO-NPs at 500 ppm concentration resulted led to a 0% disease severity among all the tested fungicides. The ZnO-NPs at 50 ppm concentration showed a 4.55 and 5.65% disease severity followed by Tebuconazole (6.33 and 7.00%), Blitox (10.20 and 11.33%) and SAAF (12.25 and 13.66%) at 7 and 14 DAI, respectively. At 100 ppm, ZnO-NP displayed 3.79 and 4.50% disease severity followed by Tebuconazole (5.25 and 5.70%), Blitox (8.75 and 8.90%), and SAAF (10.09 and 11.20%) at 7 and 14 DAI, respectively. At 250 ppm, ZnO-NP showed 1.33 and 0.93% disease severity followed by Tebuconazole (3.06 and 2.75%), Blitox (6.50 and 4.55%) and SAAF (9.00 and 5.50%) at 7 and 14 DAI, respectively. At 500 ppm concentration, ZnO-NPs completely controlled the growth of black mold. While, at this concentration, Tebuconazole showed 1.58 and 0.90% disease severity followed by Blitox (2.66 and 3.00%) and SAAF (5.50 and 6.60%) at 7 and 14 DAI, respectively. Thus, as the concentration of ZnO nanoparticles increases, it decreases the growth of microbes and at the concentration of 500 ppm growth stopped altogether.

**Table 2:** Black mold bulb disease severity (%) at pre-inoculation.

S. No.	Treatment	50 ppm		100 ppm		250 ppm		500 ppm	
		7 DAI	14 DAI	7 DAI	14 DAI	7 DAI	14 DAI	7 DAI	14 DAI
1	ZnO-NP	4.55	5.65	3.79	4.50	1.33	0.93	0.00	0.00
2	SAAF (0.25%)	12.25	13.66	10.09	11.20	8.75	9.00	5.50	6.60
3	BLITOX (0.25%)	10.20	11.33	8.75	8.90	6.50	4.55	2.66	3.00
4	Tebuconazole (0.1%)	6.33	7.00	5.25	5.70	3.06	2.75	1.58	0.90
5	Control	25.60	30.80	25.60	30.80	25.60	30.80	25.60	30.80

	At 7 DAI		At 14 DAI	
	CD (5%)	SEm±	CD (5%)	SEm±
A (Treatments)	0.26	0.09	0.20	0.07
B (DAI-days after inoculation)	0.23	0.08	0.18	0.06
A × B	0.45	0.18	0.35	0.14
CV (%)	0.04		0.03	

##### 3.3.1.4 Post-inoculation studies

**Figure 5:**
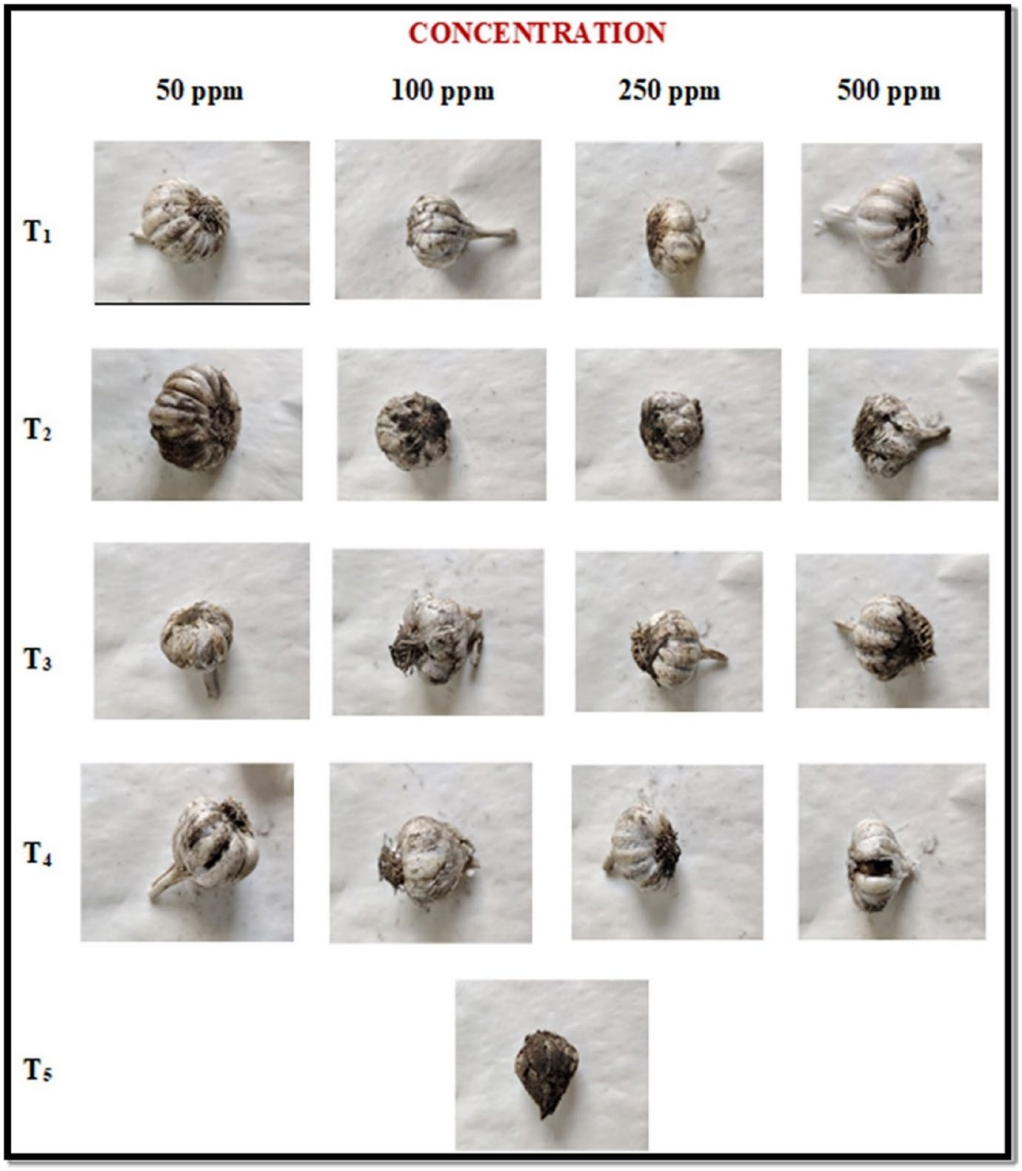
*In vivo* antifungal activities of ZnO-NPs along with commercial formulations to determine black mold bulb disease severity (%) at post-inoculation conditions. T1: ZnO-NPs; T2: SAAF 0.25%; T3: BLITOX 0.25%; T4: Tebuconazole 0.1%; T5: Control.

In post-inoculation, garlic bulb was first inoculated with spore suspension of *A. niger* and then treated with ZnO-NPs along with other selected fungicides at four different concentrations and results were summarized in Table 3 and Figure 5. Among all the tested fungicides, ZnO-NP was found best with 0 % disease severity. At 50 ppm concentration, ZnO-NPs resulted in the lowest severity (6.45 and 7.10%), followed by Tebuconazole (8.00 and 9.10%), Blitox (15.50 and 16.23%), and SAAF (18.75 and 19.66%) at 7 and 14 DAI, respectively. At 100 ppm also, ZnO-NP resulted in the lowest severity (5.60 and 5.95%), followed by Tebuconazole (7.02 and 7.90%), Blitox (13.10 and 14.07%), and SAAF (16.09 and 17.20%) at 7 and 14 DAI, respectively. At 250 ppm, ZnO-NP found best with lowest disease severity (3.30 and 2.88%) in comparison to others, followed by Tebuconazole (5.60 and 6.20%), Blitox (11.50 and 12.00%) and SAAF (14.75 and 15.30%) at 7 and 14 DAI, respectively. At higher concentration 500 ppm, ZnO-NP was found to have the lowest disease severity (1.10 and 0.90%), followed by Tebuconazole (3.92 and 4.30%), Blitox (7.76 and 8.20%), and SAAF (11.50 and 12.25%) at 7 and 14 DAI, respectively.

**Table 3:** Black mold bulb disease severity (%) at post-inoculation.

S. No.	Treatment	50 ppm		100 ppm		250 ppm		500 ppm	
		7 DAI	14 DAI	7 DAI	14 DAI	7DAI	14DAI	7 DAI	14DAI
1	ZnO-NP	6.45	7.10	5.60	5.95	3.30	2.88	1.10	0.90
2	SAAF (0.25%)	18.75	19.66	16.09	17.20	14.75	15.30	11.50	12.25
3	BLITOX (0.25%)	15.50	16.23	13.10	14.07	11.50	12.00	7.76	8.20
4	Tebuconazole (0.1%)	8.00	9.10	7.02	7.90	5.60	6.20	3.92	4.30
5	Control	35.90	42.75	35.90	42.75	35.90	42.75	35.90	42.75

	At 7 DAI		At 14 DAI	
	CD (5%)	SEm±	CD (5%)	SEm±
A (Treatments)	0.33	0.11	0.32	0.11
B (DAI-days after inoculation)	0.28	0.10	0.27	0.10
A × B	0.57	0.22	0.55	0.21
CV (%)	0.03		0.03	

## 4 Discussion

Synthesis of antifungal and antibacterial nanoparticles obtained from natural sources has emerged as a possible alternative to chemical-based medicines in recent years. These nanoparticles have been generated from natural sources. The use of conventional fungicides is widespread in the fight against fungal infections; nevertheless, these fungicides pose significant risks to the health of both humans and animals, as well as to the environment (Dinham and Malik, 2003). Another factor that has contributed to the worsening of the situation is the emergence of resistant fungus strains that include novel mutant genes. Nanobiotechnology provides a novel method for managing phytopathogenic fungi, which can help address these challenges effectively. According to Mosquera-Sanchez et al.’s research in 2020, this method has been shown to be efficient in preventing various crop illnesses caused by microorganisms. For the purpose of this investigation, we developed zinc oxide nanoparticles (ZnO-NPs) by using an environmentally friendly production method. ZnO-NPs have a number of benefits that set them apart from other nanoparticles, such as their non-toxicity, cost-effectiveness, and antifungal efficacy (Arciniegas-Grijalba et al., 2017, 2019). Moreover, they have antibacterial capabilities (Lingadurai et al., 2010; Li et al., 2009) and are a vital micronutrient in agriculture (Lingadurai et al., 2010; Prasad et al., 2017). Both of these characteristics have been demonstrated by researchers. In addition, ZnO-NPs are environmentally safe and do not cause any harm to human health, making them an excellent substitute for traditional chemical agents.

As shown in Figure 2, ZnO-NPs were generated by utilizing bacteria that were capable of tolerating zinc. These nanoparticles were then characterized using several techniques, including UV–Vis absorption spectroscopy, X-ray diffraction, particle size measurement using dynamic light scattering, zeta potential analysis, transmission electron microscopy (TEM), and SAED analysis. Several researchers (Karthik et al., 2017; Elshafie et al., 2023) recommended the use of methodologies that are comparable in terms of the characterization of nanoparticles.

The current study demonstrated a significant growth inhibitory effect of ZnO-NPs on *A. niger*, a fatal fungal infection of garlic. *In vitro* and *in vivo* studies showed that ZnO-NPs not only inhibited pathogen mycelium growth and spore germination but also controlled black mold disease severity at post-harvest conditions. Elshafie et al. (2023) studied the antifungal activities of ZnO-NPs (both green and chemically synthesized) against *A. citri* on navel orange and reported that the *in vitro* studies showed 61% (green-synthesized) and 52% (chemical-based) mycelial growth inhibition whereas *in situ* studies showed a reduced disease severity of 6.92 and 9.23% of chemical ZnO-NPs and green ZnO-NPs compared to control. Kamal et al. (2023) studied the relationship between the concentration of ZnO-NPs and growth inhibition of fungus and reported that at lower concentration (0.1 mg/mL), the NPs showed 12% growth inhibition whereas at a higher concentration (0.75 mg/mL), the ZnO-NPs showed 75% growth inhibition. Similar results were also observed in the present study to confirm that an increase in the concentration of ZnO-NPs led to a greater inhibition of fungus growth.

Abdelaziz et al. (2022) used environmentally safe myco-synthesized ZnO-NPs derived from *Penicillium expansum* ATCC 7861 to control the fungus *Fusarium oxysporum*, which causes wilt disease in eggplants. They also observed significant *in vitro* antifungal activities of ZnO-NPs, and *in vivo* tests showed a 75% reduction in disease severity. Moreover, the ZnO-NPs stimulated the recovery of eggplant by improving both morphological and metabolic parameters as compared to infected control. Similarly, Mooktida et al. (2019) investigated the effect of carboxymethyl cellulose (CMC)-coated ZnO-NPs on persimmon and tomato quality factors and observed that CMC-coated ZnO-NPs enhanced the shelf-life of fruits and delayed the disease severity by reduced respiration rate, increased fruit firmness, and a higher content of antioxidant compounds compared to the control group. Hassan et al. (2019) investigated the *in vitro* antifungal activity of green-synthesized ZnO-NPs against *Botrytis cinerea* and *Alternaria alternate* causing gray and black molds in pepper fruits and their analysis revealed that the maximum radial growth inhibition of both pathogenic fungi occurred at a by ZnO-NP concentration of 400 μg/mL. This finding is consistent with the results observed in the present study.

The generation of reactive oxygen species (ROS), such as hydrogen peroxide, superoxide radicals, hydroxyl radicals, and singlet oxygen and efflux mechanisms that release component ions, is responsible for the antifungal activity of ZnO-NPs, as stated in the recently published literature (Lipovsky et al., 2011). These ROS are responsible for causing severe damage to DNA and proteins, as well as inhibiting the development of cells. In plants, ROS are crucial signaling molecules that regulate development, responses to stress, and defense against pathogens (Thiruvengadam et al., 2024). However, high concentrations of ROS have the potential to cause oxidative stress, which may destroy lipids, proteins, and nucleic acids, thereby jeopardizing the plant growth (Huang et al., 2019). Due to the fact that ZnO-NPs have the ability to increase the formation of ROS, there has been an increase in interest in these particles (Li et al., 2020). This provides a novel technique for the control of plant diseases. The interaction between ZnO-NPs and pathogens results in the release of Zn^2+^ ions and the generation of ROS via photocatalytic and redox processes (Vitasovic et al., 2024). ROS are responsible for destructing DNA, disrupting microbial membranes, and denaturing proteins that eventually leads to the elimination of infections. Through their ability to alter the routes that ROS signaling pathways take, ZnO-NPs are able to contribute to advances in plant growth, photosynthesis, and immunological responses on the plant level. On the other hand, an excessive application of ZnO-NPs may result in increased levels of ROS, cellular damage, decreased growth, and oxidative stress. Therefore, it is of the utmost importance to optimize the concentration of nanoparticles in order to maximize the antibacterial effects while simultaneously reducing the phytotoxicity that is involved. The use of ZnO-NPs is generally considered to be an environmentally friendly alternative to conventional pesticides; nonetheless, careful concentration adjustment is necessary in order to prevent phytotoxicity. Moreover, the direct or electrostatic interaction-based contact of ZnO-NPs to fungal cell membrane disrupts its functioning and permeability of membranes (Subba et al., 2024). The Zn^2+^ ion release in suspension during the dissolution of nanoparticles inhibits the cell wall formation as it acts on *N*-acetylglucosamine (*N*-acetyl-D-glucose-2-amine) and *β*-1-3-D-glucan synthase, which are involved in the formation of chitin and other essential structure for cell wall formation (Abdelaziz et al., 2022; Kamal et al., 2023). The ZnO-NPs have been used in various advanced cutting-edge applications due to its toxicity status as Generally Recognized as Safe (GRAS) by the US Food and Drug Administration (Zhou et al., 2023). At the same time, recent studies also reported the biological toxicity of ZnO-NPs against various organisms and systems, which suggests its uses in a judicial and dose-dependent manner. The recent scientific studies revealed the importance of zinc and ZnO-NPs as an improved edible coating material due to its lower toxicity and potent antimicrobial activities with high stability and considered as a possible additive to replace hazardous chemicals and physical antibacterial materials (Awwad et al., 2020). Green-synthesized ZnO-NPs can be utilized as an effective antifungal agent against black mold fungus of garlic. These green-synthesized ZnO-NPs not only preserve but also increase shelf-life of post-harvest garlic cloves. The current study aligns with global initiatives to combat antifungal resistance, making a valuable contribution to the field of nano-agriculture and can be used as a safe alternative to chemical fungicides.

## 5 Conclusion

This study addresses the efficacy of green-synthesized zinc oxide nanoparticles (ZnO-NPs) as an antifungal agent against black mold disease in garlic caused by *A. niger*. The findings show that *Serratia* sp. may successfully synthesize ZnO-NPs, as proven by characterization experiments. *In vitro* experiments showed that 250 μg/mL ZnO-NPs had substantial antifungal efficacy, inhibiting *A. niger* mycelial growth and spore germination. Furthermore, *in vivo* post-harvest treatments demonstrated that ZnO-NPs at 500 ppm significantly inhibited disease severity in pre-inoculated garlic and reduced disease development in post-inoculated plants. These findings highlight the potential of green-synthesized ZnO-NPs as a more sustainable and effective alternative to standard chemical fungicides. Further studies are required to fully understand the potential of ZnO-NPs in agriculture, including investigations on their mechanism of action against fungi and large-scale field experiments to demonstrate their efficacy in a variety of conditions. Furthermore, creating stable, user-friendly formulations and conducting cost–benefit evaluations will help to integrate them into commercial agriculture methods. This study lays the path for less reliance on chemical fungicides while encouraging environmentally friendly crop protection alternatives.

## Data Availability

The datasets presented in this study can be found in online repositories. The names of the repository/repositories and accession number(s) can be found at: https://www.ncbi.nlm.nih.gov/, ON982549.
